# Resveratrol-Loaded Pluronic Micelles Ameliorate Scopolamine-Induced Cognitive Dysfunction Targeting Acetylcholinesterase Activity and Programmed Cell Death

**DOI:** 10.3390/ijms252312777

**Published:** 2024-11-28

**Authors:** Maria Lazarova, Miroslava Stefanova, Elina Tsvetanova, Almira Georgieva, Krasimira Tasheva, Lyubomira Radeva, Krassimira Yoncheva

**Affiliations:** 1 Institute of Neurobiology, Bulgarian Academy of Science, 1113 Sofia, Bulgaria; mira_stefanova@mail.bg (M.S.); elina_nesta@abv.bg (E.T.); almirageorgieva@gmail.com (A.G.); 2 Institute of Plant Physiology and Genetics, Bulgarian Academy of Sciences, 1113 Sofia, Bulgaria; krasitasheva@abv.bg; 3 Faculty of Pharmacy, Medical University of Sofia, 1000 Sofia, Bulgaria; l.radeva@pharmfac.mu-sofia.bg

**Keywords:** resveratrol, polymeric nanomicelles, cognitive dysfunction, neuroprotection, biogenic amines, BDNF, pCREB, rats

## Abstract

Numerous experimental studies suggest the potential for resveratrol (RVT) to be useful in the Alzheimer’s disease treatment, but its low bioavailability limits its application. This study aimed to assess the potential of resveratrol-loaded micelles as a neuronal delivery platform to protect rats from scopolamine-induced memory impairment. Resveratrol was incorporated into Pluronic micelles, and the effects of micellar (mRVT) and pure resveratrol (RVT) were compared in the model of scopolamine-induced dementia in male Wistar rats. Memory performance was assessed by a T maze test. The effect of the treatment on specific neurotransmitter levels and protein expression in the cortex and the hippocampus were evaluated biochemically. Our results revealed that the polymeric micelles were in nanoscale (approximately 33 nm) and reached 79% encapsulation efficiency. The treatment with mRVT demonstrated better spatial memory protective effect. The biochemical assays showed that mRVT in a dose of 10 mg/kg enhanced the effects of the pure drug in regard to noradrenalin neurotransmission and acetylcholinesterase inhibitory activity in the hippocampus. Furthermore, micellar resveratrol increased the cAMP-response element-binding protein expression in the cortex and hippocampus of rats as well as the Bcl2/BAX ratio, which indicated an anti-apoptotic effect in the experimental dementia model. In conclusion, our results indicated the potential of a micellar system loaded with resveratrol for neurodegenerative diseases treatment.

## 1. Introduction

Alzheimer’s disease (AD) is the most prevalent form of dementia globally, and is of great social importance. According to reports from the World Health Organization, over 55 million people worldwide are living with dementia, with nearly 10 million new cases diagnosed each year. Disorientation in time and space, communication breakdown, depression, agitation, anxiety, apathy, and psychosis are only part of the main symptoms of the disease that have profound implications for the quality of life of both patients and their family members [1,2]. Pathogenetically, AD is characterized by the aggregation of proteins with abnormal conformations, cholinergic synaptic dysfunction, monoaminergic reduction, mitochondrial impairment, oxidative stress, and apoptosis in memory-sensitive brain regions [3,4,5,6,7,8,9].

Currently, the primary approach to treating the disease focuses on symptom management, with acetylcholinesterase (AChE) inhibitors being the most effective available therapy. However, these medications are insufficient to halt the progression of the disease, and according to clinical data, at least half of AD patients do not respond to acetylcholinesterase inhibitors such as donepezil, rivastigmine, and galantamine [10]. Thus, efforts to discover effective therapeutic options for this neurodegenerative disorder are ongoing.

Resveratrol (RVT; 3,5,4′-trihydroxystilbene) is a polyphenol which is well known for its antioxidant properties. In addition, resveratrol exhibits several other significant medicinal benefits, including anti-inflammatory, anticancer, cardioprotective, and antiprotozoal effects [11,12,13,14,15]. It has been found that RVT possesses AChE-inhibitory activity in vivo [16]. Moreover, RVT has been reported to improve both cognitive and non-cognitive impairments, such as learning, memory, anxiety, and depression, and has shown promising results in Alzheimer’s disease (AD) models [17,18]. The positive effects of RVT on memory may be attributed to its ability to stimulate the expression of nerve growth factor in the hippocampus [19], to preserve brain-derived neurotrophic factor (BDNF), a key molecule for hippocampal plasticity [20], and to activate cAMP-response element-binding protein (CREB) signaling pathways [21]. Additionally, RVT has been shown to modulate neurotransmission directly. Sarubbo et al. demonstrated that RVT treatment increased serotonin (Sero), noradrenaline (NA), and dopamine (DA) levels in memory-sensitive brain regions and improved cognitive function in 20-month-old rats [22].

Despite these promising findings, clinical trials have yet to confirm the neuroprotective effects of resveratrol in AD treatment [23,24,25,26,27,28]. Its poor solubility, low bioavailability, rapid metabolism, and instability under light and enzymatic action significantly limit the potential of RVT for clinical applications. In light of these challenges, many researchers are focusing on nano-based drug delivery systems as a modern approach to drug development [29,30,31].

Pluronics^®^ are a family of amphiphilic block copolymers that have been approved by the U.S. Food and Drug Administration (FDA) and the European Medicines Agency (EMA) for use as surfactants, solubilizers, emulsifying agents, and absorption enhancers [32]. Their ability to form micelles enable them to transport various active molecules, particularly those with poor water solubility. Due to their low immunogenicity, exceptional biocompatibility, and low toxicity, they are widely utilized in pharmaceutical and clinical applications [32,33,34]. Among various types of Pluronics^®^, the copolymer abbreviated as F127 has gained considerable attention due to its wide range of biomedical applications [35]. On the other hand, its combination with Pluronic with lower HLB (e.g., Pluronic P123) provides better opportunity for loading of hydrophobic drugs in micelles. Many studies have reported the successful loading of hydrophobic drugs in Pluronic micelles and consecutive improvement of drug efficacy through micellar systems [36,37,38]. Yordanov et al. encapsulated cannabidiol in a micellar drug delivery system based on Pluronic P123 and Pluronic F127 copolymers and reported a high encapsulation efficiency of the drug (84%) [39]. Cannabidiol loading in these micelles resulted in stronger neuroprotective effects against induced oxidative stress in neuronal cells compare to pure cannabidiol. These results showed that micellar nanostructures could ensure the improvement of the biological activity of resveratrol and consequent applicability in clinical practices.

The aim of this study was to assess the potential of nanosized micellar formulations as a platform for neuronal delivery of resveratrol. For this purpose, resveratrol was incorporated in micelles formulated with biocompatible Pluronic copolymers and its possibility to protect rats from scopolamine-induced memory impairment was evaluated. The neuroprotective effect of pure (RVT) and micellar resveratrol (mRVT) on spatial working memory deficits, exploratory activity, and anxiety-like behavior of rats with scopolamine-induced dementia was investigated. In addition, the effect of mRVT on the brain acetylcholinergic (ACh), dopaminergic, noradrenergic, and serotoninergic neurotransmitter systems, AChE activity, the expression of memory-related neurotrophic (BDNF) and transcription (pCREB) factors, and the levels of B-cell lymphoma 2 (Bcl2) protein and Bcl-2-associated X (BAX) protein in the cortex and hippocampus were also monitored.

## 2. Results

### 2.1. Characteristics of Micelles

Resveratrol is highly hydrophobic (logP = 3.1) [40], which suggested its efficient incorporation in micelles. In this study, micelles were prepared from Pluronic P 123 and Pluronic F127 copolymers by applying the film hydration method. The results showed that polymeric micelles are an appropriate drug delivery system for resveratrol since a high encapsulation efficiency of 79% was achieved. The loaded micelles were characterized with a small diameter (32.76 ± 2 nm), zeta-potential of −4.00 ± 0.06 mV and index of polydispersity 0.278. The small size (less than 200 nm) and the narrow size distribution make the penetration of these systems across the blood–brain barrier possible [41]. Furthermore, the hydrophilic PEO shell could reduce the possibility of macrophage phagocytosis and hinder the formation of protein corona, leading to an increased retention time in the bloodstream [42]. These properties of the resveratrol-loaded polymeric micelles make them appropriate for parenteral application for treating neurological diseases.

**Figure 1:**
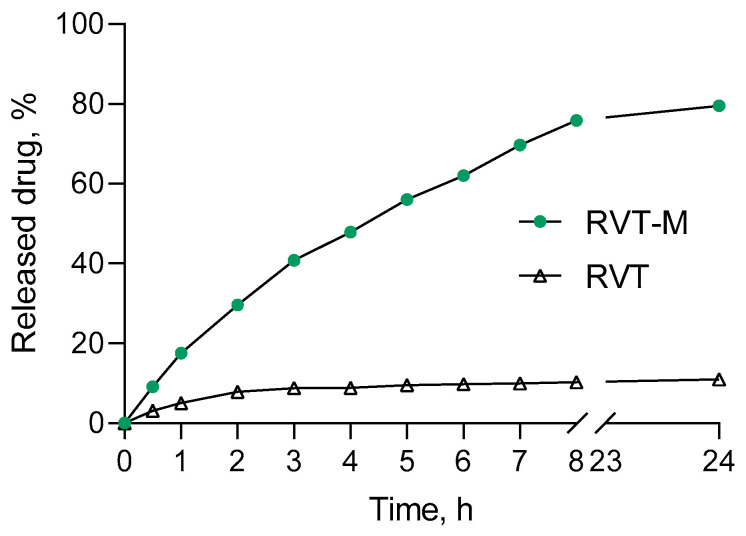
In vitro dissolution of encapsulated (mRVT) and pure resveratrol (RVT) in a phosphate buffer with pH = 7.4.

An in vitro release test showed that resveratrol was released from the micelles in a sustained manner for 24 h without an initial burst effect (Figure 1). Such a profile could enable a larger dose of the drug to reach the desired site of action and achieve a prolonged effect. Furthermore, the encapsulation of resveratrol in the micelles significantly increased its solubility. In particular, only approx. 10% of resveratrol was dissolved in the medium for 8 h, while 80% of the resveratrol was dissolved after the incorporation in the micelles. The better solubility was probably due to the transition of resveratrol from crystal to amorphous form in the micelles [43]. Thus, the loading of resveratrol into nanosized drug delivery systems could enhance its aqueous solubility, leading to improved application and effects [44].

### 2.2. Effects of Pure and Micellar Resveratrol on Spatial Working Memory

**Figure 2:**
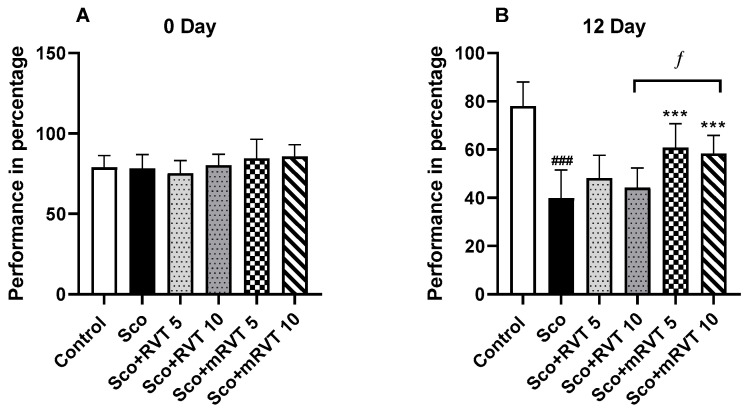
Effect of mRVT (5 and 10 mg/kg) and RVT (5 and 10 mg/kg) on rewarded spontaneous alternation behavior of rats before all treatments (**A**) and after 11 days’ scopolamine treatment (**B**) evaluated by the T maze test. Mean values ± SEM (*n* = 12 animals per group). Statistical analysis involved one-way analysis of variance (ANOVA), followed by Tukey’s multiple comparison test. Significance vs. saline-treated group: ### *p* < 0.001; significance vs. Sco-treated group: *** *p* < 0.001; significance between RVT and mRVT-treated groups: ƒ *p* < 0.05.

The animal behavior evaluated by a T maze test before and after treatments is presented in Figure 2A,B. Our experimental data indicated that, by the end of the training session, animals from all experimental groups achieved a similar rate of correct choices in the T maze test, approximately 80% (Figure 2A). The 11-day treatment with scopolamine (Sco) decreased the performance of the animals by 49% compared to the control group (*p* < 0.001, *n* = 12; Figure 2B). This indicates a reduced number of correct choices due to impaired spatial working memory. The treatment of the Sco-challenged animals with mRVT significantly increased the percentage of correct choices compared to the model group. The increase was 34% (*p* < 0.001, *n* = 12) for mRVT at a dose of 5 mg/kg and 31% (*p* < 0.001, *n* = 12) for mRVT at a dose of 10 mg/kg. In comparison, pure RVT (5 and 10 mg/kg), used as a reference, did not significantly alter the reduced number of correct choices in the Sco-challenged rats.

### 2.3. Effects of Pure and Micellar Resveratrol on Locomotion and Exploratory Activity

**Figure 3:**
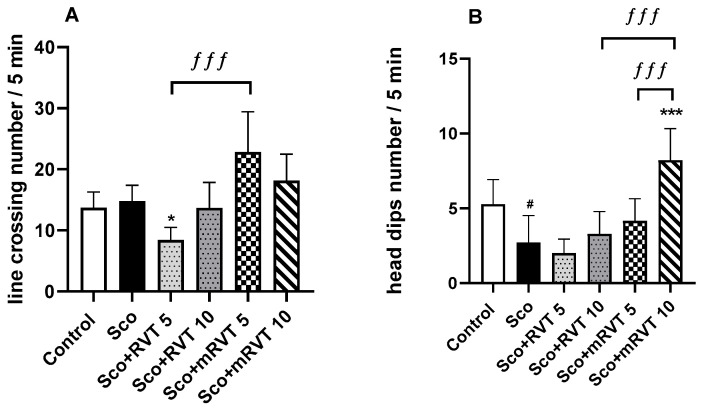
Effect of pure (RVT) and micellar resveratrol (mRVT) at concentrations of 5 and 10 mg/kg on locomotion (**A**), anxiety, and exploratory activity (**B**) of rats with scopolamine-induced memory impairment in the hole-board test. Mean values ± SEM (*n* = 12 animals per group). Statistical analysis involved one-way analysis of variance (ANOVA), followed by Tukey’s multiple comparison test. Significance vs. saline-treated group: # *p* < 0.05; significance vs. Sco-treated group: * *p* < 0.05, *** *p* < 0.001; significance between RVT and mRVT-treated groups: ƒƒƒ *p* < 0.001.

The effects of mRVT and pure RVT at doses of 5 and 10 mg/kg on locomotion, exploratory activity and anxiety-like behavior of scopolamine challenged rats are shown in Figure 3A,B. Our results revealed that 11 days of Sco-treatment did not significantly change the locomotor activity of the animals. However, it significantly decreased the number of the head dips (by 48%, *p* < 0.05, *n* = 12) compared to saline-treated animals, which was an indication for decreased exploratory activity and anxiety-like behavior.

The treatment with mRVT did not significantly change the control level of the locomotor activity of the animals at both doses, 5 and 10 mg/kg (Figure 3A), and increased the head dip numbers by 200% (*p* < 0.001, *n* = 12) compared to Sco-treated animals at a dose of 10 mg/kg (Figure 3B). In addition, the locomotor activity of the animals from the mRVT (5 mg/kg) group was 144% (*p* < 0.001, *n* = 12) higher than the activity of the animals treated with pure RVT at the same concentration. The head dip activity of the animals treated with mRVT (10 mg/kg) was 150% (*p* < 0.001, *n* = 12) higher compared to pure RVT (10 mg/kg) and 95% (*p* < 0.001, *n* = 12) higher compared to mRVT-treated (5 mg/kg) animals (Figure 3B).

The administration of pure RVT at a dose of 5 mg/kg decreased locomotor activity of dementia rats by 43% (*p* < 0.05, *n* = 12), which was an indication for motor skill problems or high levels of anxiety. The treatment with pure RVT in both concentrations did not reverse the reduced by the scopolamine head dip numbers (Figure 3B).

These results are an indication of stimulated exploratory activity and the anxiolytic effect of micellar resveratrol at a dose of 10 mg/kg.

### 2.4. Effects of Pure and Micellar Resveratrol on Brain Acetylcholinergic Neurotransmission

**Figure 4:**
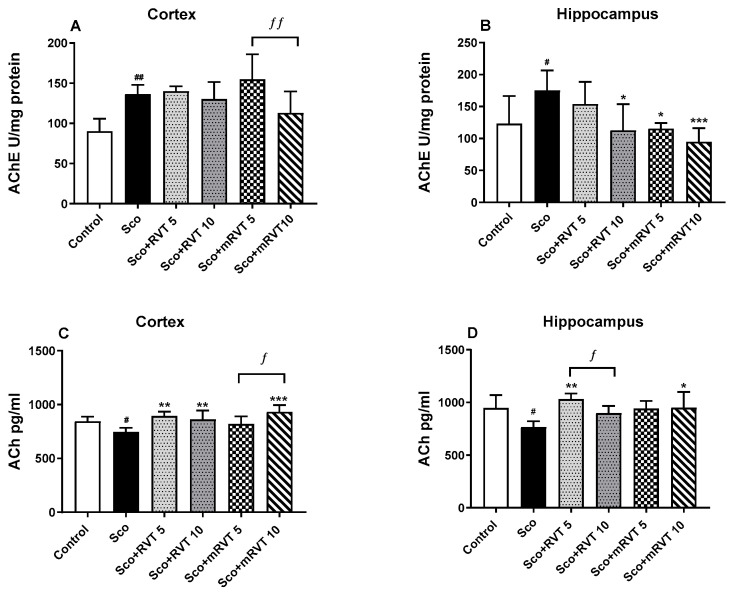
Effect of pure (RVT) and micellar resveratrol (mRVT) at 5 and 10 mg/kg concentrations on AChE activity and ACh content in cortex (**A**,**C**) and hippocampus (**B**,**D**) of rats with scopolamine-induced memory impairment. Mean values ± SEM (*n* = 6 animals per group). Statistical analysis involved one-way analysis of variance (ANOVA), followed by Tukey’s multiple comparison test. Significance vs. saline-treated group: # *p* < 0.05; ## *p* < 0.01 significance vs. Sco-treated group: * *p* < 0.05, ** *p* < 0.01, *** *p* < 0.001; significance between RVT and mRVT-treated groups: ƒ *p* < 0.05, ƒƒ *p* < 0.01.

The effects of pure and micellar resveratrol on AChE activity and ACh content in the cortex and hippocampus of Sco-treated rats are shown in Figure 4A–D and Table 1. Our results demonstrated that the Sco-treatment in the model group significantly increased AChE activity in the cortex (by 51%, *p* < 0.01, *n* = 6) and in the hippocampus (by 42% (*p* < 0.05, *n* = 6) compared to the control. The treatment with mRVT and pure RVT (5 and 10 mg/kg) did not significantly alter the scopolamine-induced increase in AChE activity in the cortex but it did reduce in the hippocampus. In the groups with pure RVT (10 mg/kg) and mRVT (5 mg/kg), the enzyme activity in the hippocampus was restored to the control levels. Further, the group treated with the higher concentration of mRVT (10 mg/kg) it was decreased by 46% (*p* < 0.001, *n* = 6) compared to the scopolamine group.

The control levels of ACh were reduced by 12% (*p* < 0.05, *n* = 6) in the cortex and by 19% (*p* < 0.05, *n* = 6) in the hippocampus after Sco-treatment. RVT and mRVT application restored Sco-induced reductions in ACh levels in both brain regions to the control levels (Figure 4C,D). The post hoc test revealed that no significant difference was observed between the effects of RVT and mRVT treatments.

### 2.5. Effects of Pure and Micellar Resveratrol on Monoamine Brain Levels

**Figure 5:**
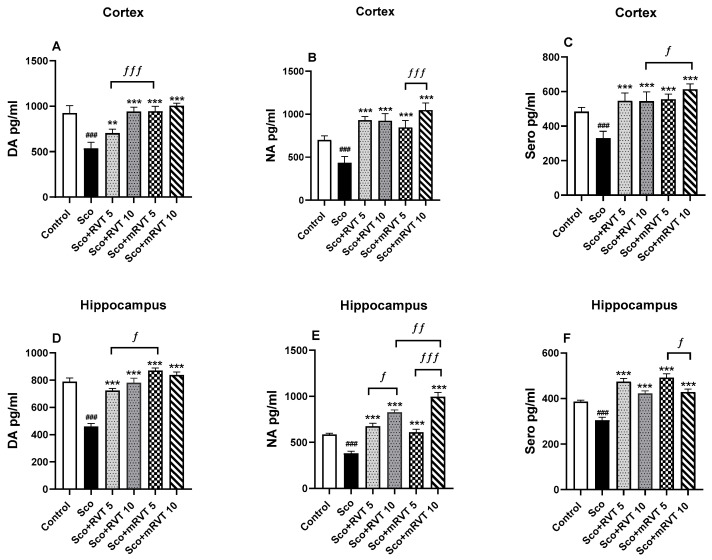
Effect of pure (RVT) and micellar resveratrol (mRVT) at 5 and 10 mg/kg concentrations on DA, NA, and Sero content in cortex (**A**–**C**) and hippocampus (**D**–**F**) of rats with scopolamine-induced memory impairment. Mean values ± SEM (*n* = 6 animals per group). Statistical analysis involved one-way analysis of variance (ANOVA), followed by Tukey’s multiple comparison test. Significance vs. saline-treated group: ### *p* < 0.001; significance vs. Sco-treated group: ** *p* < 0.01; *** *p* < 0.001; significance between RVT/mRVT-treated groups: ƒ *p* < 0.05; ƒƒ *p* < 0.01; ƒƒƒ *p* < 0.001.

The effects of pure and micellar resveratrol treatment on monoamine brain levels of Sco-treated rats are shown in Figure 5A–F and Table 2 and Table 3. The control levels of dopamine (DA), noradrenaline (NA), and serotonin (Sero) in the cortex of the experimental animals were significantly reduced after 11 days of Sco-treatment as follows: by 42% (*p* < 0.001, *n* = 6), by 38% (*p* < 0.001, *n* = 6), and by 32% (*p* < 0.001, *n* = 6), respectively. In the hippocampus, the control levels were reduced by 42% (*p* < 0.001, *n* = 6) for DA, by 35% (*p* < 0.001, *n* = 6) for NA, and by 21% (*p* < 0.001, *n* = 6) for Sero.

Our results revealed that the treatment with RVT and mRVT at doses 5 and 10 mg/kg prevented the scopolamine-induced reduction in DA and Sero brain levels, demonstrating a protective effect on these neurotransmitter systems. The post hoc test revealed that no significant difference was observed between the effects of RVT and mRVT treatments. However, the NA neurotransmission in the hippocampus was more significantly affected after the administration of the micellar drug (10 mg mRVT/kg), with NA levels increasing by 161% (*p* < 0.01, *n* = 6) compared to the model group. The post hoc test revealed significant differences between the effect of both doses of micellar resveratrol (mRVT). In the hippocampus of the mRVT-treated (10 mg/kg) animals, the NA levels were 63% (*p* < 0.001, *n* = 6) higher compared to the animals treated with 5 mg mRVT/kg and 20% (*p* < 0.01, *n* = 6) higher compared to the animals treated with pure RVT (10 mg/kg) (Figure 5B,E). In the cortex of mRVT-treated (10 mg/kg) animals the NA levels were with 24% higher (*p* < 0.001, *n* = 6) compared to the mRVT (5 mg/kg).

### 2.6. Effect of Pure and Micellar Resveratrol on the BDNF and pCREB Protein Expression

**Figure 6:**
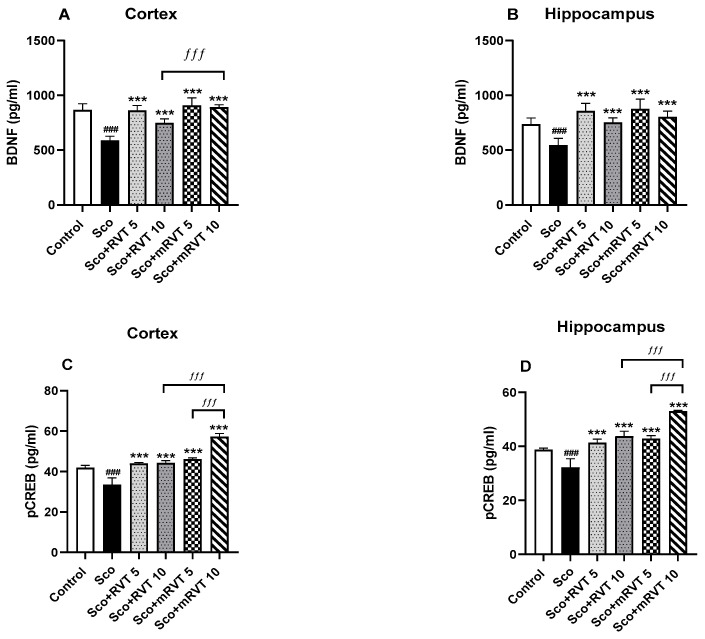
Effect of pure (RVT) and micellar resveratrol (mRVT) (5 and 10 mg/kg) on BDNF and pCREB protein expression in cortex (**A**,**C**) and hippocampus (**B**,**D**) of rats with Sco-induced memory deficit. Each column represents mean ± S.E.M. of 6 animals. Data analysis was performed using one-way ANOVA followed by Tukey’s multiple comparison test. Significance versus saline-treated group: ### *p* < 0.001; significance versus Sco-treated group: *** *p* < 0.001; significance between RVT and mRVT-treated groups: ƒƒƒ *p* < 0.001.

The BDNF and pCREB protein expression in the cerebral cortex and hippocampus of the rats were evaluate by the enzyme-linked immunosorbent assay (ELISA) method. As shown in Figure 6A,B, 11 days of Sco-treatment decreased BDNF expression in the cortex by 32% (*p* < 0.001, *n* = 6) and in the hippocampus by 26% (*p* < 0.001, *n* = 6) compared to the control. RVT and mRVT treatment preserved the control levels of BDNF protein expression in both brain structures.

**Figure 7:**
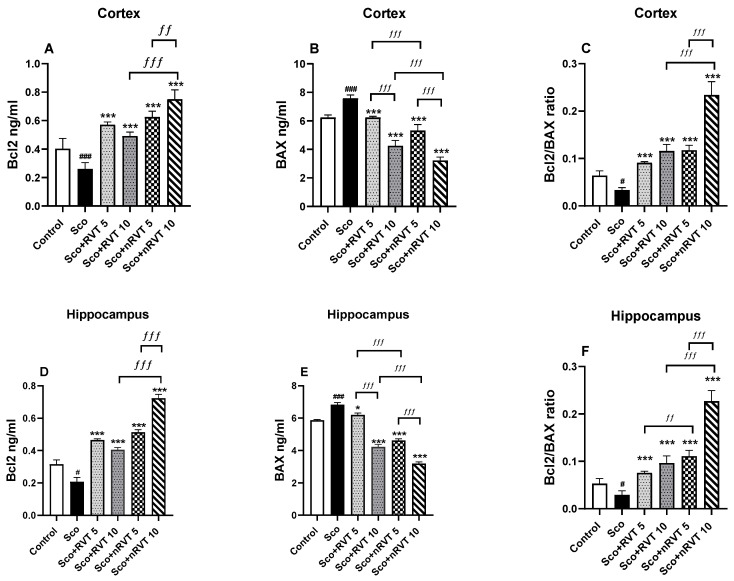
Effect of pure (RVT) and micellar (mRVT) resveratrol (5 and 10 mg/kg) on Bcl2 and BAX protein expression and Bcl2/BAX ratio in cortex (**A**–**C**) and hippocampus (**D**–**F**) of rats with Sco-induced memory deficit. Each column represents mean ± S.E.M. of 6 animals. Data analysis was performed using one-way ANOVA followed by Tukey’s multiple com-parison test. Significance versus saline-treated group: # *p* < 0.05, ### *p* < 0.001; significance versus Sco-treated group: * *p* < 0.05, *** *p* < 0.001; significance between RVT and mRVT-treated groups: ƒƒ *p* < 0.01, ƒƒƒ *p* < 0.001.

The expression of pCREB was reduced by 21% (*p* < 0.001, *n* = 6) in the cortex and by 17% in the hippocampus (*p* < 0.001, *n* = 6) after Sco-administration compared to saline treated rats (Figure 7C,D). The treatment with micellar mRVT (10 mg/kg) increased pCREB expression in the cortex by 71% (*p* < 0.001, *n* = 6) and in the hippocampus by 55% (*p* < 0.001, *n* = 6) compared to the model group. The other treatments preserved the control levels of pCREB protein expression in both brain structures. The post hoc test revealed significant differences between the effects on the pCREB expression of mRVT administration (10 mg/kg) and those of mRVT (5 mg/kg) and pure RVT (10 mg/kg) in the cortex and hippocampus. In the cortex of the animals treated with mRVT (10 mg/kg), the protein expression was increased by 24% (*p* < 0.001, *n* = 6) compared to mRVT (5 mg/kg, *p* < 0.001, *n* = 6) and by 30% (*p* < 0.001, *n* = 6) compared to pure RVT (10 mg/kg). In the hippocampus, pCREB levels in the group with mRVT (10 mg/kg) were 24% (*p* < 0.001, *n* = 6) and 21% (*p* < 0.001, *n* = 6) higher compared to mRVT (5 mg/kg) and pure RVT (10 mg/kg), respectively.

### 2.7. Effect of Pure and Micellar Resveratrol on Bcl2/BAX Protein Expression

The determination of the differences in the expression levels of the Bcl2/BAX protein apoptotic biomarkers in the cortex and hippocampus of the animals from different experimental groups are presented in Figure 7A–F. In the cortex with Sco-treatment for 11 days, the Bcl-2 expression decreased by 36% (*p* < 0.001, *n* = 6), and that of BAX increased by 22% (*p* < 0.001, *n* = 6), which reduced the control level of the Bcl2/BAX ratio by 48% (*p* < 0.05). In the hippocampus, the Bcl2 expression was decreased by 34% (*p* < 0.001, *n* = 6) and that of BAX was increased by 17% (*p* < 0.001, *n* = 6), which reduced the Bcl2/BAX ratio by 45% (*p* < 0.05, *n* = 6) compared to the control level.

RVT and mRVT treatment at doses of 5 and 10 mg/kg significantly stimulated Bcl2 and suppressed BAX expression in the cortex and hippocampus of dementia animals. However, the post hoc test revealed that the most significant impact on the Bcl2/BAX ratio was achieved with the treatment with micellar drug (mRVT, 10 mg/kg). In particular, it registered a more than 7-fold (*p* < 0.001) increase in the ratio compared to the model group, which indicated an anti-apoptotic effect.

## 3. Discussion

In this study, the potential of resveratrol-loaded Pluronic (P123/F127) micelles as a therapeutic platform for neurodegenerative diseases was evaluated. The effects of micellar resveratrol on spatial working memory deficits, exploratory activity, and anxiety-like behavior of rats with scopolamine-induced dementia were investigated and compared with that of the pure resveratrol. Additionally, their effects on the brain neurotransmitter systems (ACh, DA, NA, and Sero), AChE activity, and expression of memory-related neurotrophic factors (BDNF) and transcription factors (pCREB) were evaluated, along with the levels of Bcl2 and BAX in the cortex and hippocampus.

The scopolamine model of dementia is a valuable tool for exploring therapeutic interventions, as its intraperitoneal injection induces Alzheimer’s disease-like alterations, including deficits in novel object recognition, spatial, associative, and recognition memories [45,46,47,48,49,50,51,52,53,54], cholinergic and monoaminergic systems dysfunction, and disrupts redox homeostasis by depleting antioxidant defenses [3,4,5,47,52,55,56,57]. Additionally, reductions in biomarkers of synaptic integrity such as BDNF and pCREB [49,50,51,52,54,58] and a decrease in the Bcl2/BAX ratio, indicating increased apoptosis levels [52,59], were also observed after Sco-treatment. All these changes led to cognitive and memory deficits in experimental animals.

Resveratrol is a plant-derived polyphenol with potential therapeutic benefits for Alzheimer’s disease treatment, but it shows limited efficacy in clinical settings mainly because of poor water solubility [30]. In this study, resveratrol was encapsulated in mixed Pluronic (P123/F127) micelles aiming to improve its solubility and intracellular transport. Some studies have already reported an improvement in drug solubility and drug activity after its incorporation in Pluronic micelles [60,61,62]. In this study, an in vitro release test revealed that the resulting resveratrol-loaded micelles provided better drug solubility. Furthermore, the mean diameter size of the micelles (approximately 33 nm) would improve the intracellular transport.

A T maze test was applied aiming to compare the effect of micellar and pure resveratrol (5 and 10 mg/kg) on spatial working memory in rats with Sco-induced dementia. Spontaneous alternation in a T maze paradigm is used to measure the exploratory behavior of animals and reflects their spatial working memory [63,64,65,66]. Spontaneous alternation occurs when a rodent chooses the opposite arm of the T maze from the one in its previous trial, demonstrating its ability to remember which arm it visited last. The memory for the previous visit is stored in its spatial working memory [67]. The results from the T maze test in this study (Figure 2) demonstrated that the Sco-treatment significantly decreased the number of correct choices in the model group compared to the control, indicating a substantial impairment in spatial working memory. At the same time, the micellar resveratrol in both concentrations (5 and 10 mg/kg) increased the number of correct choices made by the Sco-challenged rats, whereas the pure resveratrol in the same concentrations did not significantly change the score of the model group. Our findings revealed that micellar resveratrol has the potential to ameliorate scopolamine-induced cognitive deficits more effectively than the pure drug used as a referent, which could be due to the improved solubility and absorption.

However, since spontaneous alternation in rodents depends on optimal levels of anxiety [68,69], we compared the effects of pure and micellar resveratrol on anxiety-like behavior using the hole-board test (Figure 3). In addition, the exploratory activity and locomotion of the animals were also monitored [70]. Our results showed that scopolamine treatment did not significantly alter the control levels of locomotor activity of rats, suggesting that the worsened behavioral performance observed in this group was not related to impaired motor skills. However, the previously documented reduction in exploratory activity and anxiety-like effects of the muscarinic antagonist, evidenced by a decreased number of head dips compared to the controls, were also observed [49,71]. We found that among all resveratrol applications in the present work only micellar resveratrol in 10 mg/kg combined unchanged control levels of locomotor activity of the animals with increased head dips numbers, indicating an increased exploratory activity and a lower level of anxiety. Based on these observations, we suggest that micellar resveratrol at a dose of 10 mg/kg may enhance the reported ameliorative effects of the drug on anxiety and depression [72].

As a next step, the effect of micellar and pure resveratrol was compared regarding AChE activity and brain levels of some relevant neurotransmitters in the cortex and hippocampus of the experimental animals from all groups (Figure 4). AChE is an enzyme that breaks down the neurotransmitter acetylcholine in the synaptic cleft and remains a major target in Alzheimer’s treatment. Its inhibition helps maintain higher levels of acetylcholine in the brain and is a standard mechanism to slow disease progression [73]. The Sco-treatment resulted in an increase in AChE activity and a decrease in ACh and monoamine levels in the brain, which aligns with our previous observations [48,51,54,74].

Previous studies reported the AChE-inhibitory activity of resveratrol [16] and its stimulatory effect on the brain monoaminergic neurotransmission [75]. In this research, resveratrol treatments (both pure and micellar) in dementia animals demonstrated AChE-inhibitory activity in the hippocampus and an ACh and monoaminergic preservation effect in both observed brain structures. This finding aligns with the observations of Foudah and colleagues, who reported that the oral administration of RVT in rats with scopolamine-induced memory impairment improved cognitive function by the selective suppression of AChE activity in the hippocampus [16]. The biochemical results highlighted that micellar resveratrol at a dose of 10 mg/kg exhibited the strongest AChE-inhibitory activity in the hippocampus and potential to increase norepinephrine levels in the same region. Thus, the results revealed the potential of the micelles to enhance the effects of the drug.

Further, the effects of micellar and pure resveratrol were compared based on their influence on the expression levels of BDNF and pCREB. BDNF is a neurotrophic factor that in the adult brain regulates both excitatory and inhibitory synaptic transmission and activity-dependent plasticity [76,77]. It is essential for memory processes and long-term memory formation at the hippocampal and cortical synapse [78]. BDNF signaling is closely related to the CREB, a transcription factor that binds to the promoter regions of many neuronal genes associated with learning, memory, and synaptic plasticity [79,80,81,82]. The dysfunction of CREB and reduced BDNF levels in the brains of both AD patients and transgenic mice have been reported [83,84,85]. It is assumed that the CREB–BDNF signaling pathway may represent a promising target for the development of novel compounds with memory-enhancing properties. It is known that resveratrol preserves BDNF [20,86] and activates CREB signaling pathways [21]. Several studies using various animal models of depression and cognitive impairment have reported that resveratrol treatment alleviates cognitive deficits and reductions in BDNF and pCREB/CREB levels, likely through the modulation of neuroinflammation [87,88] and the activation of the Sirt1/miR-134 pathway [89]. In our study, the effects of pure and micellar resveratrol were comparable and demonstrated neuroprotection in the cortex and hippocampus (Figure 6). Related to pCREB, only the micellar resveratrol in 10 mg/kg increased the protein expression in both brain structures. The other treatments preserved the control levels of pCREB.

Apoptosis is a tightly regulated form of programmed cell death. The main components of the apoptotic signaling network are two Bcl-2 family members, Bcl-2 and BAX [90]. Bcl-2, a key anti-apoptotic protein of the Bcl-2 family, promotes cell survival by preventing apoptosis triggered by various stimuli [91]. In contrast, BAX is a pro-apoptotic protein, which promotes apoptosis by translocation into the mitochondrial membrane and by facilitating cytochrome *c* release to propagate downstream apoptotic signal transduction [92]. The ratio between pro-apoptotic and anti-apoptotic proteins from the Bcl-2 family members determines the fate of cells [93,94]. Since alterations in the expression of these proteins have been reported in vulnerable neurons in AD, apoptosis has been proposed to explain the cell loss observed in this neurological disorder [95,96].

In this study, we used scopolamine to assess the sensitivity of cortical and hippocampal neurons in rats treated with resveratrol (pure and micellar form) to apoptosis. Our findings showed that scopolamine treatment increased Bax expression and decreased Bcl-2 levels in the cortex and hippocampus, reducing the Bcl-2/Bax ratio, which indicated the induction of neuronal apoptosis (Figure 7). As neuronal apoptosis in the cortex and hippocampus significantly impacts learning and memory processes [97,98], our results corroborated the cited studies.

The anti-apoptotic properties of resveratrol (RVT) and its ability to influence the hippocampal expression of Bax, caspase-3, and Bcl-2 have been previously reported [99]. Additionally, resveratrol has been shown to activate upstream pathways, such as the PI3K/Akt signaling cascades [100]. The PI3K/Akt pathway plays a critical role in neuronal cell survival [101], regulating the expression of pro-survival proteins, including NF-κB, CREB, Bcl-2, and Bax [102,103,104,105]. Our results revealed that the treatments of the Sco-challenged rats with pure resveratrol in both concentrations and micellar resveratrol in the lower concentration (5 mg/kg) restored the Bcl-2/Bax ratio to control levels in both brain regions. More important, the micellar resveratrol at 10 mg/kg significantly increased the ratio beyond the control. This fact suggested that the expression of Bax and Bcl-2 was most significantly affected by this treatment, indicating a strong anti-apoptotic effect.

In conclusion, our research demonstrated that mRVT treatment enhanced the pharmacological activity of the pure drug. This was reflected in its stronger AChE-inhibitory activity, increased noradrenaline neurotransmission, the activation of BDNF/CREB signaling, and pronounced anti-apoptotic effects in the cortex and hippocampus of rats with experimental dementia. These improvements contribute to its superior neuroprotective properties and enhanced efficacy in improving learning and memory.

## 4. Materials and Methods

### 4.1. Materials

Trans-resveratrol was provided from Sigma Chemical Co. (Schnelldorf, Germany). Pluronic^®^ F 127 (PEO_101_PPO_56_PEO_101_) and Pluronic^®^ P 123 (PEO_20_PPO_70_PEO_20_) were obtained from BASF (Ludwigshafen, Germany). The following rat ELISA kits, ordered by Elabscience, Houston, TX, USA, were used: ACh (cat. no E-EL-0081); DA (cat. no E-EL-0046); NA (cat. no E-EL-0047); Sero (cat. no E-EL-0033); rat BDNF ELISA kit (cat. no. E-EL-R1235); rat Bcl-2 ELISA kit (cat. no. E-EL-R0096); rat BAX ELISA kit (cat. no. E-EL-R0098). Rat pCREB ELISA kit (cat. no. SL1344Ra) was purchased from Sunlong Biotech Co., Ltd., Hangzhou, China.

### 4.2. Formulation and Characterization of Resveratrol-Loaded Micelles

Resveratrol was loaded in Pluronic micelles via the film hydration method. The ratio between resveratrol and polymers was 1:10 (*w*/*w*). Briefly, resveratrol was added to a methanol solution of Pluronic F127 and P123 (ratio between the polymers 1:1 wt/wt). Thereafter, the solvent was evaporated at room temperature, the obtained film was redispersed in distilled water, and the dispersion was filtered (0.2 µm). The filter was rinsed with ethanol (50%), and the amount of non-loaded resveratrol in the rinsed fraction was determined by UV-spectrophotometry at 306 nm (Thermo Fisher Scientific, Waltham, MA, USA). The concentration of the non-loaded resveratrol was calculated according to a standard curve (2–10 µg/mL, r > 0.9996). The following equations were used for determining the loading degree (LD) and encapsulation efficiency (EE):

LD (mg/mL) = (Total amount of RVT − Non-loaded RVT)/Volume of RVT loaded dispersion(1)

EE (%) = (Total amount of RVT − Non-loaded RVT) × 100/Total amount of RVT(2)

The in vitro release test was carried out via dialysis in a phosphate buffer (pH 7.4) containing 10% ethanol. Micellar dispersion (2 mL) was poured in a membrane (10,000 MWCO, Spectrum Labs, San Francisco, CA, USA) that was further immersed in 80 mL of the buffer and gently shaken in a water bath at 37 °C (IKA Labortechnik HS-B20, Staufen, Germany). At time intervals of 1 h, buffer samples were withdrawn from the acceptor phase, and equivalent volume of fresh medium was returned in order to maintain sink conditions. The concentrations of the released resveratrol were determined UV spectrophotometrically.

### 4.3. Animals

The study used male Wistar rats (200–250 g) (Erboj Laboratories, Sofia, Bulgaria). The animals were housed under standard laboratory conditions (25 ± 3 °C, 12 h light/dark cycle) with full-time access to food and water. The experimental protocol started after a five-day habituation period. 

### 4.4. Groups and Administration

The rats were divided into 6 groups (*n* = 12 per group) and injected intraperitoneally (i.p.) as follows: control group treated with normal saline (0.5 mL/100 g); scopolamine (model) group treated with scopolamine hydrobromide water solution (Sco, 2 mg/kg, i.p) for 11 consecutive days [49,74,106,107]; Sco+RVT 5 mg/kg, Sco+RVT 10 mg/kg; Sco+mRVT 5 mg/kg and Sco+mRVT 10 mg/kg groups treated simultaneously with Sco and RVT (30% hydroethanolic solution) or mRVT respectively. The doses 5 and 10 mg/kg were considered from a previous study [108]. The RVT and mRVT were applied 60 min before the Sco-treatment.

### 4.5. Behavioral Observations

**Figure 8:**
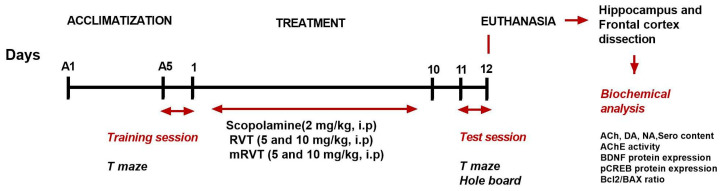
Timeline of the experiment.

To evaluate memory disturbance, locomotion, and exploratory behavior, the animals from all groups were subjected to T maze and hole-board tests. Behavioral tests were conducted from 9 a.m. to 12 p.m. in a dimly lit room at the end of the experiment (Figure 8).

#### 4.5.1. T Maze Test

The alteration in spatial working memory of the rats was assessed by measuring changes in spontaneous alternation behavior in the T maze test [109]. The apparatus (Stoelting Co., Wood Dale, IL, USA) was a standard T maze [110] with one start runway (50 cm long and 10 cm wide) and two finish arms (40 cm long, 10 cm wide). The maze was made of gray, non-reflective plexiglass.

The T maze behavioral test began with habituation, followed by training and test sessions. The habituation period lasted 2 days, during which the animals explored the maze. The training session spanned 5 to 6 days before treatments, aiming to train the animals to alternate between the two finish arms. Goal entries were defined as the rat placing all four paws in the arm. A choice was considered correct if the rat selected the arm opposite to the one chosen in the previous T maze trial. Every correct choice was rewarded with food. During training, all animals underwent ten trials a day with a 5 min inter-trial interval. The training period ended after control animals made 80% correct choices on two consecutive days. The test session was carried out on the 12th day after the first treatment, recording the number of correct entries. Each animal performed 10 trials with a five-minute interval between each trial, starting with a forced first attempt (on the right). The data are presented as a percentage, calculated by dividing the number of correct choices by the total number of trials minus 1, then multiplying by 100.

The choices during the training and test sessions were always rewarded with food at the end of the arm. For motivational purposes, during the entire training session, the animals were fed a limited diet (5 g/100 g body weight). Before the test session, the animals were left without food for 24 h, with only water to drink. The rats were habituated to all procedures and tested by a single tester.

#### 4.5.2. Hole-Board Test

The hole-board test apparatus is a square platform measuring 50 × 50 cm. The floor of the apparatus is raised, containing 16 circular holes (3 cm/diameter) located symmetrically on the surface and divided into nine equal squares. To assess locomotor activity, rats were placed in the center of the board, and the number of squares traversed within 5 min was recorded. To assess exploratory activity and anxiety-like behavior, the number of head dips within 5 min was counted [66].

### 4.6. Brain Tissue Dissection

One hour following the behavioral tests, animals were humanely euthanized via mild CO_2_ inhalation and decapitated. The whole brains were taken out and flushed with ice-cold saline. The prefrontal cortex and hippocampus were isolated and homogenized in phosphate-buffer saline (PBS) for ACh and monoaminergic determination. The homogenates were centrifuged at 5000× *g* for 10 min.

The cortical and hippocampal AChE activity was assayed as described previously in [111]. The protein content was measured by the method described in [112].

The acetylcholine (Ach) and monoamines (DA, NA, and Sero) content in tissue lysate was determined by an enzyme-linked immunosorbent assay after homogenization following the manufacturer instructions. The concentrations were measured using a microplate reader at 450 nm. The results were reported in picograms per milliliter (pg/mL).

The concentrations of BDNF, pCREB, Bcl2, and BAX in the frontal cortex and hippocampus of rats were quantified by the ELISA method, following the manufacturer instructions. The protein concentrations were measured using a microplate reader at 450 nm. The results were reported in picograms per milliliter (pg/mL).

### 4.7. Statistical Analysis

Shapiro–Wilk and Kolmogorov–Smirnov normality tests were applied to examine the individual distribution of parameters. Only normally distributed data were subjected to further parametric evaluation. Data are expressed as the mean ± standard error of the mean (SEM). All graphs and the statistical analysis were performed using the Prism 8.0 software program (GraphPad Software, Inc., San Diego, CA, USA). The data from the T maze test, hole-board test, and ELISA method were analyzed by one-way analysis of variance (ANOVA) followed by Tukey’s post hoc comparison test. A difference between the groups was considered significant at ^#^ *p* < 0.05, ^##^ *p* < 0.01, ^###^ *p* < 0.001 vs. saline-treated group, and * *p* < 0.05, ** *p* < 0.01, *** *p* < 0.001 vs. Sco-treated group.

## 5. Conclusions

This research demonstrated that the encapsulation of resveratrol in polymeric micelles resulted in a better protective spatial working memory effect compared to the pure drug in rats with scopolamine-induced memory impairment. Furthermore, the micellar form of resveratrol at a dose of 10 mg/kg increased the levels of noradrenaline and inhibited acetylcholinesterase activity in the rat hippocampus to a higher degree compared to the pure resveratrol. In addition, the micellar resveratrol increased the pCREB protein expression in both brain regions. The significant increase in the Bcl2/BAX ratio after treatment with the micellar resveratrol (10 mg/kg) indicated an anti-apoptotic effect in the experimental dementia model, suggesting that the micelles could improve resveratrol’s bioavailability. Considering that the micellar resveratrol is in an aqueous formulation, we consider this system promising for further investigation as a delivery platform.

## Figures and Tables

**Table 1:** The effects of mRVT and pure RVT on the concentrations of acetylcholine (Ach) in frontal cortex and hippocampus of dementia rats.

Group	Dose (mg/kg)	Ach in Frontal Cortex (pg/mL)	Ach in Hippocampus (pg/mL)
Control	-	844.99 ± 14.89	947.14 ± 50.02
Sco	2	747.35 ± 12.45 #	767.11 ± 22.27 #
RVT	5	892.66 ± 20.79 **	1030.37 ± 26.70 **
RVT	10	862.15 ± 36.62 **	897.93 ± 26.08
mRVT	5	819.57 ± 31.92	942.19 ± 32.35
mRVT	10	931.30 ± 25.91 ***	949.34 ± 61.49 *

Table values are expressed as mean ± S.E.M. with units of pg/g for 6 rats in each group. Data analysis was performed using one-way analysis of variance (ANOVA), followed by Tukey’s multiple comparison test. Significance vs. saline-treated group: # *p* < 0.05; significance vs. Sco-treated group: * *p* < 0.05, ** *p* < 0.01, *** *p* < 0.001.

**Table 2:** The effects of mRVT and pure RVT on the concentrations of neurotransmitters DA, NA, and Sero in frontal cortex of dementia rats.

Group	Dose mg/kg	Frontal Cortex (pg/mL)		
		DA	NA	Sero
Control		924.23 ± 33.63	700.00 ± 19.66	484.53 ± 9.35
Sco	2	535.68 ± 27.42 ###	435.00 ± 29.97 ###	330.64 ± 16.14 ###
RVT	5	703.78 ± 21.52 **	930.00 ± 21.21 ***	546.21 ± 23.13 ***
RVT	10	941.78 ± 19.66 ***	923.33 ± 34.12 ***	545.08 ± 21.55 ***
mRVT	5	943.36 ± 25.19 ***	846.00 ± 35.44 ***	555.86 ± 12.99 ***
mRVT	10	1005.83 ± 10.37 ***	1048.33 ± 33.60 ***	613.51 ± 12.54 ***

Table values are expressed as mean ± S.E.M. with units of pg/g for 6 rats in each group. Data analysis was performed using one-way analysis of variance (ANOVA), followed by Tukey’s multiple comparison test. Significance vs. saline-treated group: ### *p* < 0.001; significance vs. Sco-treated group: ** *p* < 0.01, *** *p* < 0.001.

**Table 3:** The effects of micellar (mRVT) and pure drug (RVT) on the concentrations of neurotransmitters DA, NA, and Sero in hippocampus of dementia rats.

Group	Dose mg/kg	Hippocampus (pg/mL)		
		DA	NA	Sero
Control		788.38 ± 27.24	788.38 ± 27.24	386.82 ± 5.40
Sco	2	459.72 ± 22.00 ###	381.66 ± 22.71 ###	304.82 ± 12.28 ###
RVT	5	723.77 ± 15.96 ***	675.00 ± 32.27 ***	473.67 ± 13.90 ***
RVT	10	780.46 ± 34.06 ***	826.66 ± 24.72 ***	422.24 ± 11.35 ***
mRVT	5	869.28 ± 19.85 ***	610.00 ± 33.16 ***	491.82 ± 16.89 ***
mRVT	10	836.90 ± 22.27 ***	995.00 ± 47.66 ***	428.88 ± 12.27 ***

Table values are expressed as mean ± S.E.M. with units of pg/g for 6 rats in each group. Data analysis was performed using one-way analysis of variance (ANOVA), followed by Tukey’s multiple comparison test. Significance vs. saline-treated group: ### *p* < 0.001; significance vs. Sco-treated group: *** *p* < 0.001.

## Data Availability

Data is contained within the article.
